# Plasma pTau181 predicts cortical brain atrophy in aging and Alzheimer’s disease

**DOI:** 10.1186/s13195-021-00802-x

**Published:** 2021-03-29

**Authors:** Cécile Tissot, Andréa L. Benedet, Joseph Therriault, Tharick A. Pascoal, Firoza Z. Lussier, Paramita Saha-Chaudhuri, Mira Chamoun, Melissa Savard, Sulantha S. Mathotaarachchi, Gleb Bezgin, Yi-Ting Wang, Jaime Fernandez Arias, Juan Lantero Rodriguez, Anniina Snellman, Nicholas J. Ashton, Thomas K. Karikari, Kaj Blennow, Henrik Zetterberg, Etienne De Villers-Sidani, Philippe Huot, Serge Gauthier, Pedro Rosa-Neto, for the Alzheimer’s Disease Neuroimaging Initiative

**Affiliations:** 1 The McGill University Research Centre for Studies in Aging, Douglas Hospital, McGill University, 875 La Salle Blvd – FBC room 3149, Montreal, QC H4H 1R3 Canada; 2 Translational Neuroimaging Laboratory-McGill University, Montreal, QC Canada; 3 Douglas Hospital Research Centre, Verdun, QC Canada; 4 Department of Epidemiology and Biostatistics, McGill University, Montreal, QC Canada; 5 Department of Psychiatry and Neurochemistry, Institute of Neuroscience and Physiology, The Sahlgrenska Academy, University of Gothenburg, Gothenburg, Sweden; 6 Wallenberg Centre for Molecular and Translational Medicine, University of Gothenburg, Gothenburg, Sweden; 7 King’s College London, Institute of Psychiatry, Psychology & Neuroscience, Maurice Wohl Clinical Neuroscience Institute, London, UK; 8 NIHR Biomedical Research Centre for Mental Health & Biomedical Research Unit for Dementia at South London & Maudsley NHS Foundation, London, UK; 9 Clinical Neurochemistry Laboratory, Sahlgrenska University Hospital, Mölndal, Sweden; 10 UK Dementia Research Institute at UCL, London, UK; 11 Department of Neurodegenerative Disease, UCL Institute of Neurology, London, UK; 12 Center for Research on Brain, Language and Music, McGill university, Montreal, QC Canada; 13 Neurodegenerative disease groups, Montreal Neurological Institute, Montreal, QC Canada; 14 Le Centre intégré universitaire de santé et de services sociaux (CIUSSS) de l’Ouest-de-l’Île-de-Montréal, Montreal, QC Canada; 15 Department of Neurology and Neurosurgery, Psychiatry and Pharmacology and Therapeutics, McGill University, Montreal, Canada

**Keywords:** Plasma pTau181, Neurodegeneration, Voxel-based morphometry, Alzheimer’s disease

## Abstract

**Background:**

To investigate the association of plasma pTau181, assessed with a new immunoassay, with neurodegeneration of white matter and gray matter cross-sectionally and longitudinally, in aging and Alzheimer’s disease.

**Methods:**

Observational data was obtained from the Alzheimer’s Disease Neuroimaging Initiative, in which participants underwent plasma assessment and magnetic resonance imaging. Based on their clinical diagnosis, participants were classified as cognitively unimpaired and cognitively impaired. Linear regressions and linear mixed-effect models were used to test the cross-sectional and longitudinal associations between baseline plasma pTau181 and neurodegeneration using voxel-based morphometry.

**Results:**

We observed a negative correlation at baseline between plasma pTau181 and gray matter volume in cognitively unimpaired individuals. In cognitively impaired individuals, we observed a negative association between plasma pTau181 and both gray and white matter volume. In longitudinal analyses conducted in the cognitively unimpaired group, plasma pTau181 was negatively correlated with gray matter volume, starting 36 months after baseline assessments. Finally, in cognitively impaired individuals, plasma pTau181 concentrations were negatively correlated with both gray and white matter volume as early as 12 months after baseline, and neurodegeneration increased in an incremental manner until 48 months.

**Conclusions:**

Higher levels of plasma pTau181 correlate with neurodegeneration and predict further brain atrophy in aging and Alzheimer’s disease. Plasma pTau181 may be useful in predicting AD-related neurodegeneration, comparable to positron emission tomography or cerebrospinal fluid assessment with high specificity for AD neurodegeneration.

## Background

Advances in quantification of biofluids made possible the detection of Alzheimer’s disease (AD) pathophysiological processes in peripheral plasma. It was recently demonstrated that ultra-sensitive tau phosphorylated at threonine-181 (pTau181) in plasma [1–4] provides an inexpensive way to determine the presence of brain neurofibrillary tangles in vivo. Recent studies of plasma pTau181 [1, 5–7] were successful at differentiating AD from other neurodegenerative conditions and presented a strong correlation with pTau181 concentrations in the cerebrospinal fluid (CSF) [1]. Although the associations between CSF and biomarkers of neurodegeneration have been extensively described, little is known regarding plasma pTau181 and its cross-sectional and longitudinal associations with neurodegeneration of white matter (WM) and gray matter (GM). It was however observed that plasma pTau181 levels correlate with lower gray matter volume in the precuneus and temporal lobe of mild cognitive impairment (MCI) and AD participants [6]. Moreover, the novel method to assess plasma pTau181, which is used in the following analyses, has been shown to predict a 1-year cognitive decline and hippocampal atrophy along the AD spectrum [1]. In the current investigation, we examine whether plasma pTau181 correlates with neurodegeneration assessed via voxel-based morphometry (VBM) cross-sectionally, and longitudinally, over a maximum of a 4-year period. We conducted analyses in cognitively unimpaired (CU) and cognitively impaired (CI) individuals, including MCI and AD participants, who are part of the Alzheimer’s Disease Neuroimaging Initiative (ADNI). We hypothesize that plasma pTau181 levels are associated with baseline neurodegeneration of WM and GM, as well as predict subsequent atrophy.

## Methods

### Study participants

Data used in the preparation of this article was obtained from the Alzheimer’s Disease Neuroimaging Initiative (ADNI) database (adni.loni.usc.edu). The ADNI was launched in 2003 as a public-private partnership, led by Principal Investigator Michael W. Weiner, MD. The primary goal of ADNI has been to test whether serial magnetic resonance imaging (MRI), positron emission tomography (PET), other biological markers, and clinical and neuropsychological assessment can be combined to measure the progression of mild cognitive impairment (MCI) and early Alzheimer’s disease (AD). Data used was downloaded on June 20, 2020. Each ADNI site received approval from their Ethics Board to conduct the study. Written consent was obtained from all the research participants.

The ADNI inclusion/exclusion criteria are described in detail at www.adni-info.org (accessed June 2020). The individuals underwent MRI scans, an assessment of plasma pTau181, and a neuropsychological evaluation. Participants were considered CU when they had a Clinical Dementia Rating (CDR) of 0 and MCI when they obtained a CDR of 0.5, while individuals suffering from dementia due to AD were considered such with a CDR of 1 or higher and met the standard diagnostic criteria for probable AD [8]. The CI group was composed of both MCI and AD individuals. Participants who had no objective evidence of cognitive impairment but reported subjective cognitive decline were analyzed together with the CU individuals, as per the National Institute of Aging-Alzheimer’s Association [9]. Baseline diagnosis was used for statistical analyses.

**Fig. 1:**
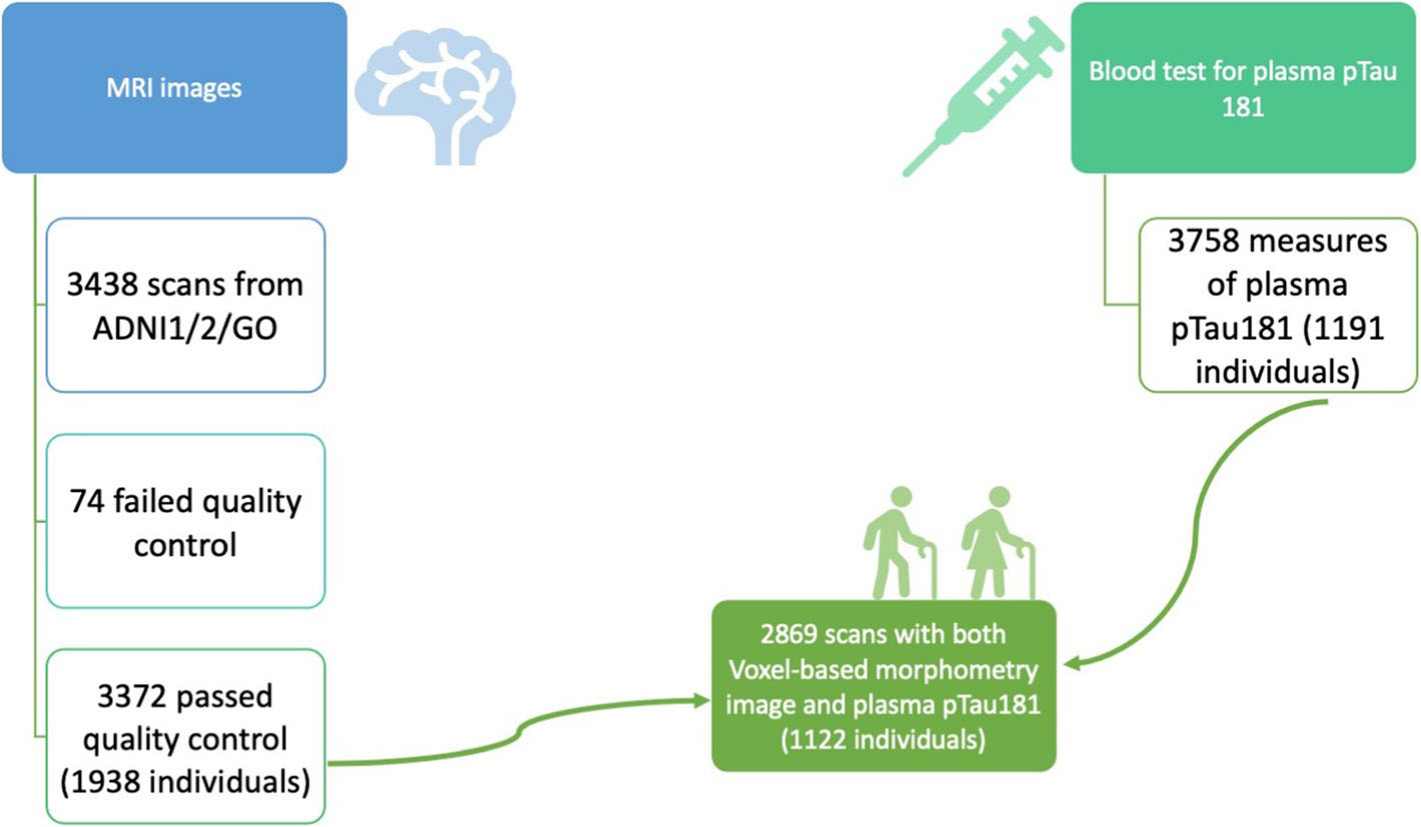
Sample selection from ADNI. 3438 MRI were available from participants, 74 of them failed quality control, ending up to 3364 available scans to study. Similarly, 3758 participants had available results from plasma ptau181. Combined together, we had 2869 available scans and plasma pTau181 results which dates of assessment were within 6 months

A detailed description of the sample selection can be found in Fig. 1. The number of individuals at each time point can be found in Table 1.

**Table 1:** Number of individuals at each time point

Time points	Number of participants
BL	1122 (384 CU and 738 CI)
V12	786 (238 CU and 548 CI)
V24	605 (240 CU and 365 CI)
V36	177 (51 CU and 126 CI)
V48	179 (73 CU and 106 CI)

### Imaging analyses

Pre-processed 1.5-T and 3-T T1-weighted MRI scans were downloaded from the ADNI database (adni.loni.usc.edu; for pre-processing details, see [10]). Anatomical images were segmented into probabilistic gray matter (GM) and white matter (WM) maps using the SPM12 segmentation tool. Each GM and WM probability map was then non-linearly registered (with modulation) to the ADNI template using DARTEL [11] and smoothed with a Gaussian kernel of full width half maximum (FWHM) of 8 mm. All images were visually inspected to ensure proper alignment to the ADNI template.

### Plasma measurements

Plasma pTau181 was measured using a clinically validated in-house assay described previously [1]. Plasma pTau181 was measured on Simoa HD-X instruments (Quanterix, Billerica, MA, USA) in April 2020 at the Clinical Neurochemistry Laboratory, University of Gothenburg, Mölndal, Sweden, by scientists blinded to participants’ clinical information. Plasma pTau181 data was collected over 47 analytical runs. Assay precision was assessed by measuring three different quality control samples at the start and end of each run, resulting in within-run and between-run coefficients of variation of 3.3–11.6% and 6.4–12.7% respectively. Out of 3762 ADNI samples, four were removed due to inadequate volumes. The remaining 3758 all measured above the assay’s lower limit of detection (0.25 pg/ml), with only six below the lower limit of quantification (1.0 pg/ml), which were excluded from the study.

### Statistical analyses

R statistical software package (version 4.0.0) was used to perform the nonimaging statistical analyses. Plasma pTau181 results were log-transformed to meet the requirements of parametric statistics. We first conducted *t*-test (continuous variables) and chi-square tests (categorical variables) for demographics.

We also conducted linear mixed-effect (LME) regression models, using the *lme4* package, in order to compare the progression of plasma pTau181 in each diagnostic group over time. The LME included plasma pTau181 as the dependent variable and the interaction between time and group as the independent variable. The covariates were sex and age at baseline. To accommodate the correlation arising from multiple measurements on the same participant, we also included a random intercept. The 95% confidence intervals were estimated based on the estimated fitted value across the distribution from 1000 simulations of the model that includes all variations. All tests mentioned previously were two-sided with a statistical significance level of *P* < .05.

For brain imaging, we conducted linear models (LM) and LME at the voxel level using VoxelStats [12]. We studied the associations between log-transformed plasma pTau181 and GM and WM images. Firstly, we investigated the relationship between log-transformed plasma pTau181 and VBM images cross-sectionally for each diagnostic group (CU and CI) separately, adjusting for sex and age at baseline. Additionally, we investigated the longitudinal associations between baseline plasma pTau181 and VBM images. The LME included VBM images as the dependent variable and the interaction between time and baseline plasma pTau181 as the independent variable. They were adjusted for sex and age at baseline, as well as random intercept. The model was performed in CU and CI individuals separately. Finally, to infer disease progression at each visit, we used the same statistical model using the follow-up visit (time) as a categorical variable. We used random field theory [13] (RFT) to correct all imaging results for multiple comparisons. Exploratory analyses were also conducted correcting for random slopes.

## Results

### Demographics

**Fig. 2:**
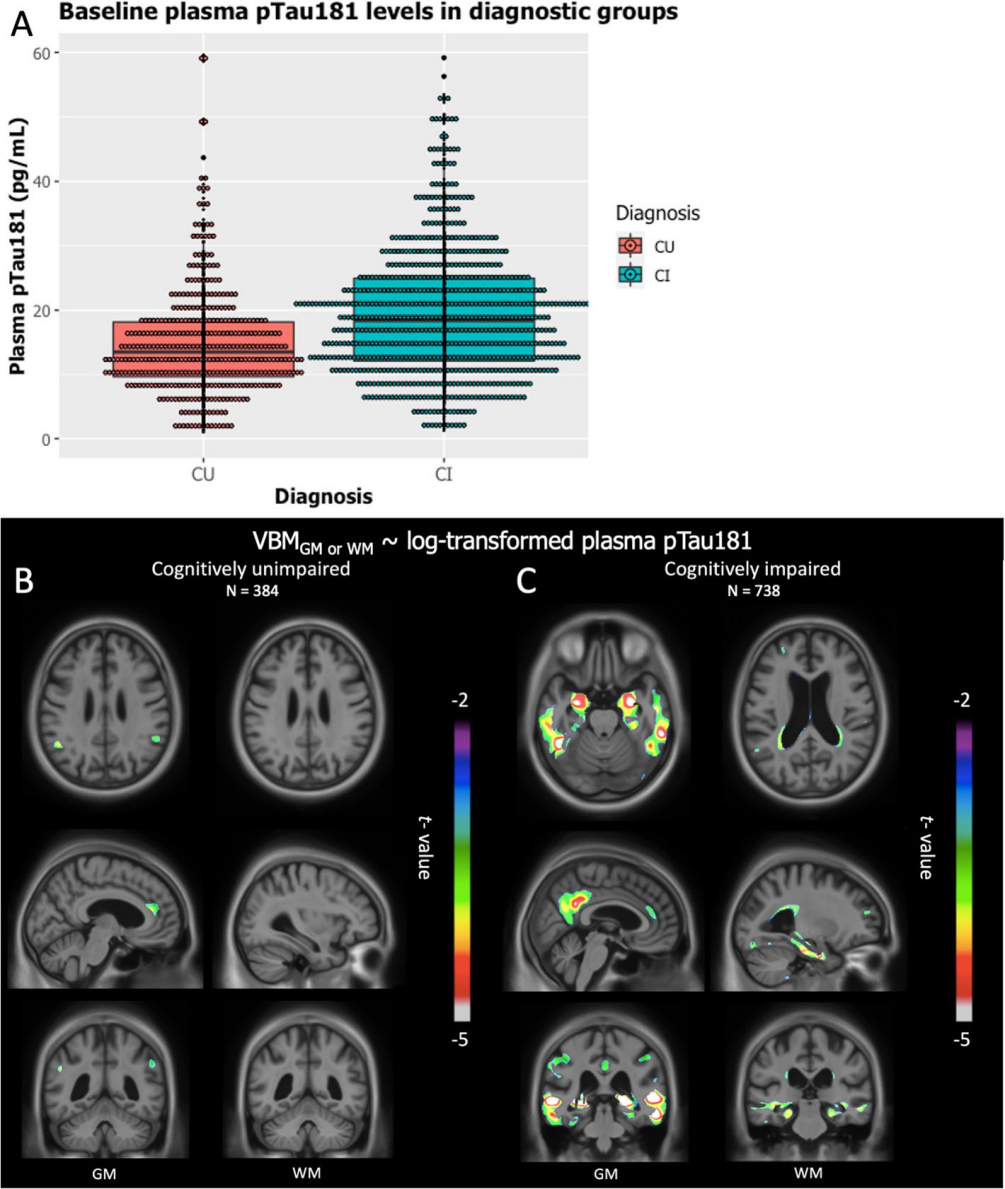
Associations between plasma tau 181 and neurodegeneration predominate in CI individuals. **a** Mean of log-transformed plasma pTau181 depending on the diagnostic group. **b** CU individuals showed a negative correlation with GM in the anterior cingulate and occipital gyrus. However, no correlation was found with WM. **c** CI individuals presented a negative correlation with GM in the precuneus, anterior cingulate, medial, and lateral temporal gyrus. WM decrease was found in the corpus callosum and the temporal lobe

Demographic information can be found in Table 2. The sample included a total of 1122 individuals, among which 384 were CU and 738 were CI (539 MCI and 199 AD). The average follow-up time was 22 ± 11.63 months. There was no statistically significant difference between the groups in terms of age. However, there were statistically significant differences in terms of sex, MMSE scores, and the plasma pTau181 levels between the CU and CI individuals. The CI group was composed predominantly of males and showed lower MMSE scores and higher plasma pTau181 levels. For the CU group, the mean of plasma pTau181 levels was 15.48 ± 10.02 pg/mL, while for the CI, it was 19.80 ± 10.75 pg/mL (Fig. 2a).

**Table 2:** Characteristics of participants included in the study. The sample was composed of 384 cognitively unimpaired individuals, and 738 cognitively impaired, among which 539 were diagnosed with mild cognitive impairment and 199 with probable Alzheimer’s disease

Characteristics	CU	CI
Number of subjects	384	738
Age (mean, SD) in years	74.40 (6.50)	73.61 (7.94)
Females (*n*, %)	205 (53%)^‡^	311 (42%)^‡^
MMSE score (mean, SD)	29.06 (1.23)^†^	26.36 (3.68)^†^
Plasma pTau181 (mean, SD)	15.48 (10.02)^†^	19.80 (10.75)^†^

^‡^Statistical difference between groups (*P* < 0.05)

^†^Statistical difference between groups (*P* < 0.001)

### Regional association between plasma pTau181 with WM and GM volume predominate in CI

Cross-sectional analysis in the CU group revealed that plasma pTau181 was not associated with WM volume. Nevertheless, there was a negative relationship with GM volume. This association was found in the anterior cingulate and the lateral occipital gyrus (Fig. 2b).

In CI individuals, we discovered strong negative associations between plasma pTau181 and GM as well as WM volumes. Correlations between the blood-based biomarker and GM were observed in the precuneus, the anterior cingulate, and the medial and lateral temporal gyrus. WM and plasma pTau181 associations were found in the corpus callosum and the temporal lobe (Fig. 2c). R-maps of the cross-sectional analyses can be found in Supplementary Figure 1.

### Plasma pTau181 predicts subsequent GM and WM volume decline

**Fig. 3:**
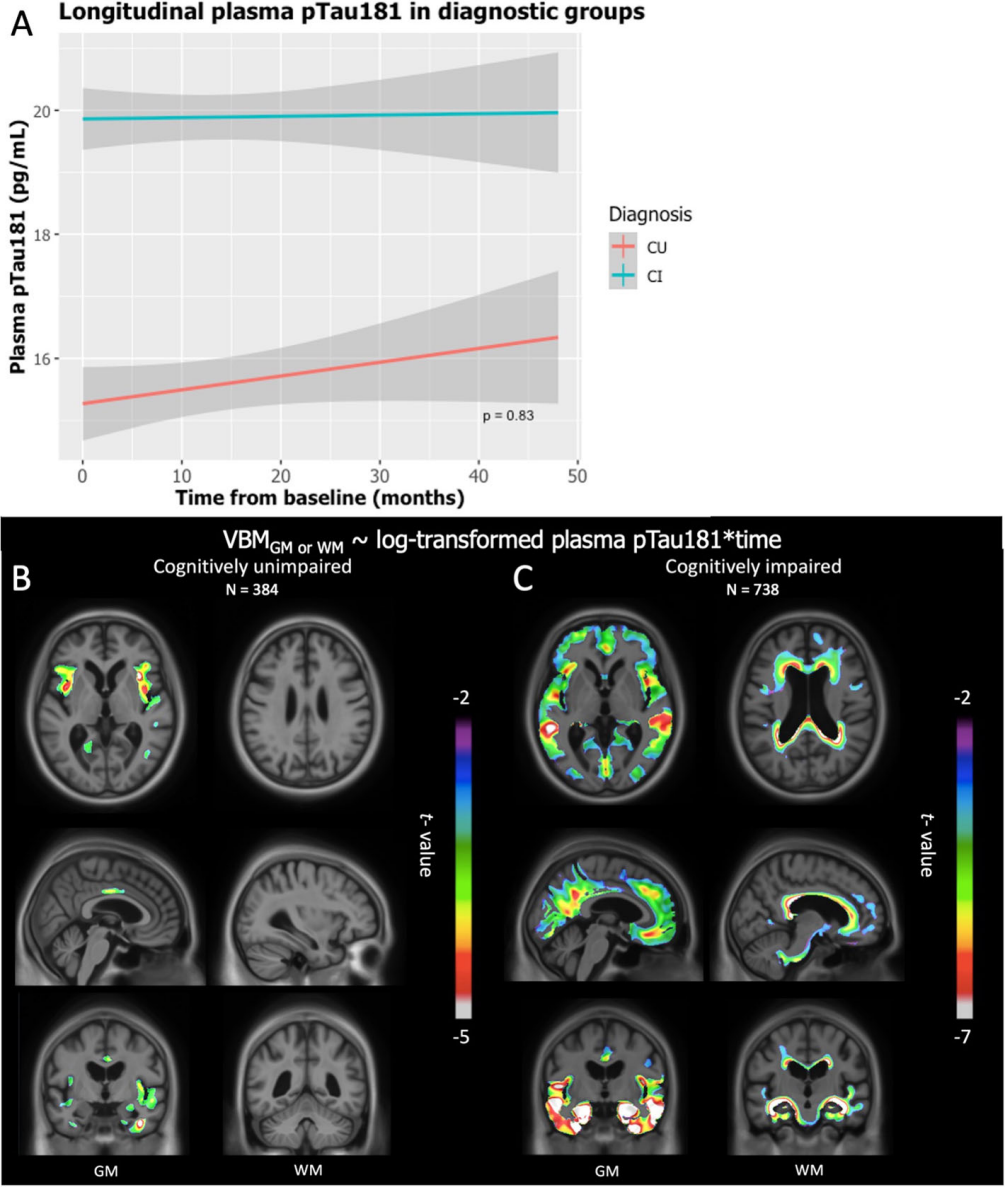
Association between plasma pTau181 and rate of brain atrophy in AD-related areas. **a** There is no significant difference between the rates of change of plasma pTau181 between CU and CI individuals. **b** CU individuals presented a negative correlation between plasma pTau181 and GM in the temporal lobe, precuneus, and anterior cingulate cortex. No correlation was found with WM. **c** CI individuals showed a negative correlation with GM in the precuneus, frontal cortex, and temporal lobe. WM results showed a negative correlation in the corpus callosum, the temporal, and the frontal lobes

There was no significant difference in the rate of change of plasma pTau181 between CU and CI groups over a period of 48 months (*P* = 0.16) (Fig. 3a). Individual changes can be seen in Supplementary Figure 2. LME models conducted in the CU group showed a negative association between plasma pTau181 and GM volume in the temporal lobe, the precuneus, and the anterior cingulate cortex. Similar analyses with WM volume changes did not survive correction for multiple comparisons (Fig. 3b). In the CI individuals, negative associations between plasma pTau181 and GM volume were observed in the precuneus and the frontal cortex, with even stronger associations in the temporal area. Plasma pTau181 and WM volume showed a negative association in the corpus callosum and the frontal and temporal lobes (Fig. 3c). When correcting for random slopes, the results were identical.

### Negative associations between plasma pTau181 and GM or WM volume spread over time

**Fig. 4:**
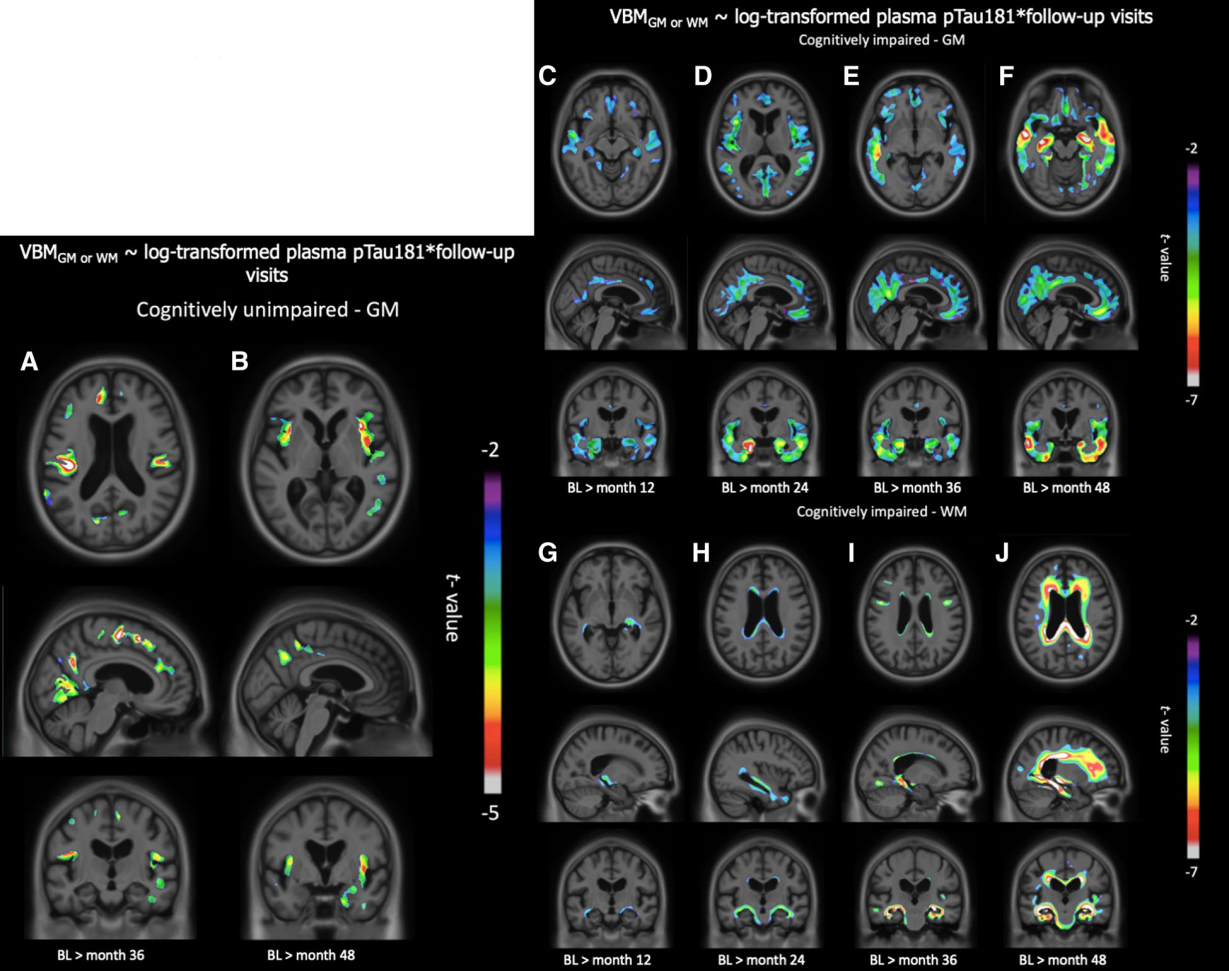
Negative associations between plasma pTau181 and GM or WM volume progressed over time. **a** CU individuals showed GM degeneration 36 months after baseline, in the precuneus, medial frontal, anterior cingulate, and temporal lobe. **b** Similar regions were affected 48 months after baseline. **c** The CI group presented GM degeneration 12 months after baseline, in the medial frontal, precuneus, posterior cingulate, and temporal lobe. **d** At month 24, similar regions were affected spreading to the medial occipital cortex. **e**, **f** At months 36 and 48, it spread even further in the medial frontal, temporal, and posterior regions of the brain. **g** In the CI group, 12 months after baseline, there was a negative correlation between WM and plasma pTau181 in the temporal lobe. **h** At month 24, it started spreading to the corpus callosum. **i** At month 36, the WM tracts in the frontal and occipital lobe were affected. **j** At month 48, most of the WM in the brain seemed affected

Among the CU individuals, negative correlations between plasma pTau181 with GM were observed at the 36-month FU (Fig. 4a) and the 48-month FU (Fig. 4b) in the precuneus, insula, medial frontal, anterior cingulate, and finally temporal lobe. Comparatively, there was no significant negative relationship with WM volume.

In the CI group, areas in which GM volume negatively correlated with plasma pTau181 progressively expanded from 12- to 48-month FU. As early as 12-month FU, pTau181 and GM volume correlations were restricted to the medial frontal, precuneus, posterior cingulate, and temporal lobe (Fig. 4c). At 24-month FU, these negative correlations also included the medial occipital cortex (Fig. 4d). From 36- (Fig. 4e) to 48-month FU (Fig. 4f), these associations embraced the whole medial frontal cortex, the precuneus, the medial occipital, and the temporal lobes. Plasma pTau181 and WM volume associations similarly progressed over time; these correlations were initially confined to the vicinity of the choroidal fissure as well as the temporal horn of the lateral ventricle at 12-month FU (Fig. 4g). Subsequently, they encompassed the lateral periventricular WM at 24-month FU (Fig. 4h), including frontal and occipital lobes at 36-month FU (Fig. 4i) to finally embrace the whole WM 48 months after baseline (Fig. 4j).

## Discussion

In summary, we found that plasma pTau181 was associated with GM loss in both CU and CI groups, while its associations with WM loss were observed only in CI cross-sectionally. In CU individuals, plasma pTau181 predicted GM degeneration in AD-related regions starting 36 months after baseline. In CI, plasma pTau181 predicted an incremental degeneration, in both GM and WM, which started in the typical AD-related brain regions, and encompassing the cortex and WM globally 4 years after the first assessment. The VBM changes seen as early as 12 months after baseline support the hypothesis that plasma pTau181 predicts imminent neurodegeneration.

Cross-sectional analyses suggested that measures of tau phosphorylation with plasma pTau181 inform about GM degeneration among CU individuals. The cross-sectional associations occurred only in areas well known to be affected early in the AD process, such as the cingulate cortex, in which early amyloid deposition is often observed [14]. In CU, the anterior cingulate degeneration has been linked to complex attentional deficits and is known to be impaired along the clinical spectrum of AD [15]. Other studies also presented anterior cingulate atrophy as a predictor of conversion to dementia due to AD in memory-impaired individuals [16], suggesting the region is affected before the onset of cognitive symptoms.

In CI individuals, we observed a strong negative correlation between GM loss and plasma pTau181 in regions commonly affected in AD [17], such as the medial temporal lobe, the precuneus, and the anterior cingulate. The medial temporal region, which encompasses the hippocampus, is well known to be related to early atrophy in AD [18, 19]. The associations found between plasma pTau 181 and precuneus atrophy also corroborates the finding that this region is often impaired at the early stages of AD [17, 20]. Similarly, as in the CU individuals, the CI group presented GM degeneration in the anterior cingulate, giving further support to the idea that neurodegeneration in this specific region is associated with tau hyperphosphorylation. Furthermore, we observed associations between plasma pTau181 and WM damage along the temporal lobe as well as the corpus callosum. Temporal WM atrophy has been consistently linked to aging and early AD [21]. The region is known to connect a network of memory-related areas. The associations between temporal WM and plasma pTau 181 might indicate a vulnerability of these WM tracts to hyperphosphorylation. Finally, the periventricular WM was also shown to correlate with plasma pTau181 in the CI group. Ventricular dilation and periventricular WM fibers have been presented as a biomarker of neurodegeneration [22]. Taken together, the cross-sectional analyses performed in this study provide evidence that high plasma pTau181 indicates neurodegeneration in brain regions vulnerable to early AD pathology.

Our longitudinal analyses revealed that plasma pTau181 predicted GM degeneration in various brain regions known to be affected in AD [17]. Particularly, the temporal lobe and posterior cingulate have shown atrophy in people with high plasma pTau181 baseline levels in CU individuals. Atrophy and tau deposition in both regions are well correlated with deficits of memory formation and retrieval in AD [23]. Functional alterations of the cingulate cortex imposed by AD pathophysiology have been proved to forecast dementia 2 years later [24]. Interestingly, we showed that plasma pTau181 predicts neurodegeneration in those specific regions, making it a possible earlier predictor of upcoming brain atrophy and possibly cognitive changes. By contrast, we did not observe associations between plasma pTau181 and longitudinal WM degeneration in cognitively unimpaired individuals. It is possible that WM is affected later in the disease process as compared to GM. Nevertheless, in CI individuals, we observed a strong negative correlation between plasma pTau181 and both GM and WM degeneration longitudinally. Plasma pTau181 predicted subsequent medial temporal, as well as precuneus, medial frontal, and medial occipital degeneration. Those brain regions are known to be included in the Default Mode Network, crucial for cognitive tasks [25]. AD pathophysiologies in these brain regions are also associated with memory dysfunction [26] and are known to accumulate neurofibrillary tangles [17]. It is well described that there is a decrease in the Default Mode Network connectivity along the continuum of normal aging to dementia [26]. The default mode alterations are associated with deficits in memory retrieval and envisioning the future, among others [25]. Regarding WM, plasma pTau181 predicted degeneration more specifically in the temporal and periventricular WM. WM abnormalities in these regions are considered crucial for memory formation and retrieval [21] and are also seen as markers of neurodegeneration in AD [22]. In WM, tau hyperphosphorylation can potentially come from glia or merely be associated to GM tau [27]. These results indicate that high plasma pTau181 in symptomatic cases harbingers AD-related neurodegeneration.

Further longitudinal analyses conducted at each FU MRI revealed how plasma pTau181 predicted neurodegeneration in both CU and CI groups. Although no effects were observed in the WM of CU individuals, plasma pTau181 predicted cortical neurodegeneration at month 36 after baseline in the temporal GM, the medial frontal cortex, the anterior cingulate, and the precuneus. These regions are known to be vulnerable to early AD pathology and important for memory [16–18]. The same analyses with time as an ordinal variable were conducted in the CI group; we observed an incremental degeneration of both WM and GM, resembling Braak staging [17]. At 12-month FU, we observed that plasma pTau181 was related to GM degeneration in the medial temporal, medial frontal, and precuneus regions. In the course of the 4 years post-baseline, we observed that the GM degeneration increased and spread more broadly to the entire cortex. At month 48, the medial temporal cortex, medial frontal cortex, and precuneus were significantly associated with plasma pTau181 concentrations. Similarly, the WM deterioration progresses with time. At month 12, alterations were restricted to the temporal lobe, affected early in AD, while at month 48, the frontal WM and the corpus callosum were heavily impaired. In the CI group, plasma pTau181 was able to predict imminent brain atrophy, which broadened incrementally to affect the entire brain. Taken together, these results suggest that plasma pTau181, in addition to being a cost-effective and scalable marker of future tau hyperphosphorylation, incorporates information regarding present and upcoming neurodegeneration.

The current findings between plasma pTau181 and brain atrophy corroborate previous research on CSF and plasma pTau181 [6, 28]. Higher levels of phosphorylated tau predicted neurodegeneration in the medial temporal and periventricular WM among other regions in aging and AD [28, 29]. In CI individuals, plasma pTau181 was correlated with degeneration of GM in the precuneus and temporal lobes [6]. It also predicted atrophy in the hippocampal region along the AD spectrum [1]. Blood-based biomarkers, particularly pTau181, or hyperphosphorylated tau, represent an important step in facilitating disease diagnosis and possibly patient management. Moreover, blood-based biomarkers could serve to enrich a population for clinical trial selection. The last decade has focused on the research of biomarkers useful in AD [30], leading to the development of the current assay to assess plasma pTau181. The present study provides evidence of an added value associated with the new immunoassay for plasma pTau181. The higher the plasma pTau181 levels, the higher is the probability of co-existing neurodegeneration and the higher the likelihood of developing brain degeneration in the subsequent years. Indeed, among the CU individuals, it predicted cortical atrophy in AD-related brain regions 3 years later, while in the CI individuals, it forecasted imminent atrophy of both GM and WM as soon as 12 months later, with a progressive degeneration spreading all over the brain.

Other plasma markers have been studied in AD, such as neurofilament light (NfL) and amyloid-β (Aβ), which are also promising candidates for blood-based biomarkers [31]; however, both have shown some disadvantages. Indeed, NfL is a non-specific biomarker, as it has been related to other neurodegenerative conditions [32, 33] and is related to aging. Cerebral amyloidosis can be found in CU individuals [34] as well as a proportion of non-AD dementias. Furthermore, there is a significant production of peripheral Aβ expression [35] which may not be related to the AD process. Nevertheless, the current assay for plasma pTau181 is highly specific for AD [1] with the advantage to inform about coexistent and future neurodegeneration.

### Limitations

The main strength of the study is to provide insights regarding levels of plasma pTau181, using a novel assessing method, and regional brain atrophy in a large cohort of well-characterized individuals. The assay was previously shown to be AD specific, when compared to other neurodegenerative conditions [1]. However, further research is required in larger cohorts with different tauopathies. There are various methodological limitations in the study, such as the fact that not all individuals had follow-up assessments every 12 months along the 4-year period post-baseline. Demographic differences were also present among groups, which were however accounted for in the statistical models. In ADNI [36], a multi-center cohort, and in other single-study cohorts [1], plasma pTau181 levels have been shown to increase with disease severity. Individuals presenting amyloid positivity had significantly higher levels of plasma pTau181 as compared to CU individuals [36]. In our analyses, the CU and CI groups present a significant difference in plasma pTau181 concentrations, albeit a certain overlap. It is thought to be due to the combination of MCI and AD together in the CI group, and the non-stratification depending on the amyloid status. Apart from these limitations, the new plasma pTau181 immunoassay could be proposed as a simple and scalable way to diagnose AD as it is highly associated with amyloidosis and neurofibrillary tangles and, according to our analysis, predict future brain atrophy in aging and dementia due to AD. To conclude, we provide evidence that this novel test for plasma pTau181 has a strong negative correlation with brain atrophy patterns typical of AD and can also help predict subsequent neurodegeneration in aging and dementia due to AD.

## Conclusion

The current study showed, in a large cohort of well-characterized individuals, that plasma pTau181 correlates with neurodegeneration at baseline and predicts further brain atrophy. Indeed, CU individuals mainly present GM atrophy, while CI individuals display both GM and WM damage. The incremental patterns of neurodegeneration involve brain regions related to AD pathologies. PET and CSF assessments being more expensive and not readily available, plasma pTau181 may then be a useful, cost-effective, and scalable biomarker to predict AD-related neurodegeneration, with high specificity for AD.

## Supplementary Information

**Additional file 1: Supplementary Figure 1.** Correlations maps (R-maps) of cross-sectional analyses, in both CU and CI groups. **Supplementary Figure 2.** Longitudinal changes of plasma pTau181 in each individual. **Supplementary Figure 3.** Longitudinal plasma pTau181 changes among cognitively unimpaired and cognitively impaired individuals, stratified by Aβ status.

## Data Availability

All data can be found on adni.loni.usc.edu.

## Abbreviations

AD: Alzheimer’s disease
ADNI: Alzheimer’s Disease Neuroimaging Initiative
Aβ: Amyloid-β
CDR: Clinical Dementia Rating
CI: Cognitively impaired
CSF: Cerebrospinal fluid
CU: Cognitively unimpaired
FU: Follow-up
GM: Gray matter
LM: Linear models
LME: Linear mixed effects
MCI: Mild cognitive impairment
MRI: Magnetic resonance imaging
NfL: Neurofilament light
PET: Positron emission tomography
pTau: Phosphorylated tau
RFT: Random field theory
VBM: Voxel-based morphometry
WM: White matter
