# Supplementary material for: Plasma pTau181 predicts cortical brain atrophy in aging and Alzheimer’s disease
Source: Alzheimers Res Ther. 2021 Mar 29;13:69. doi: 10.1186/s13195-021-00802-x (PMC8008680; doi:10.1186/s13195-021-00802-x)
Supplement: Supplementary file 1 — Additional file 1: Supplementary Figure 1. Correlations maps (R-maps) of cross-sectional analyses, in both CU and CI groups. Supplementary Figure 2. Longitudinal changes of plasma pTau181 in each individual. Supplementary Figure 3. Longitudinal plasma pTau181 changes among cognitively unimpaired and cognitively impaired individuals, stratified by Aβ status. [file 13195_2021_802_MOESM1_ESM.docx]

**Supplementary material**

***Supplementary figures***:


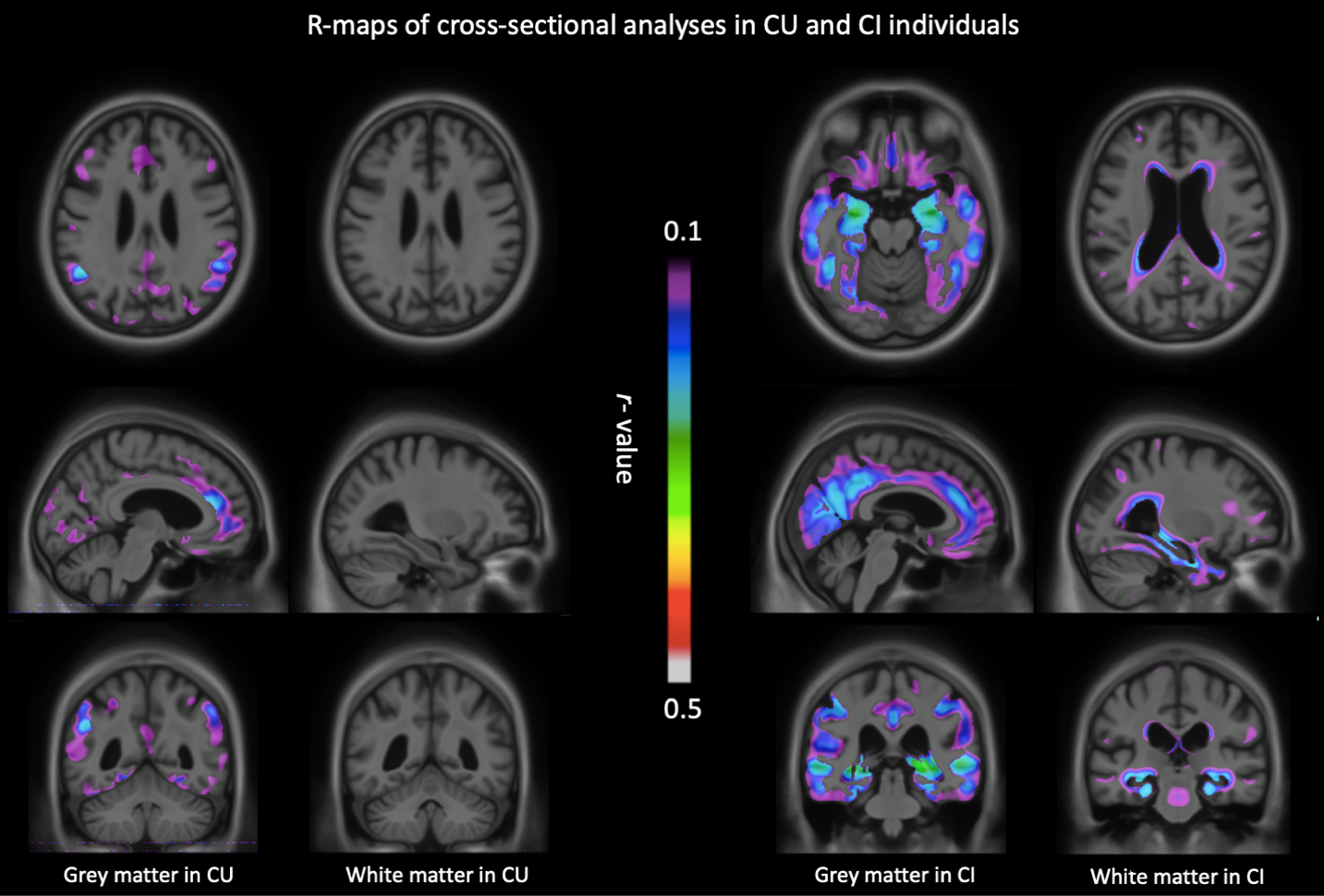


Supplementary Figure 1: Correlations maps (R-maps) of cross-sectional analyses, in both CU and CI groups.


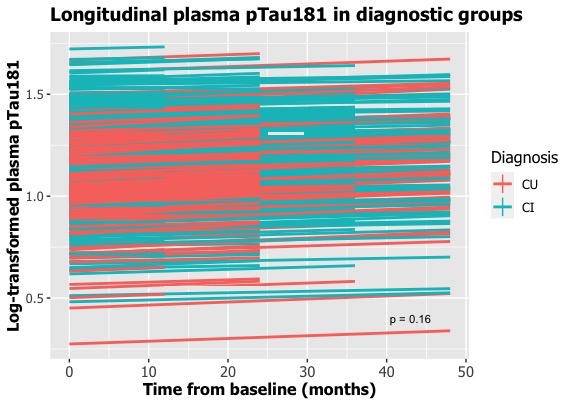


Supplementary Figure 2: Longitudinal changes of plasma pTau181 in each individual.


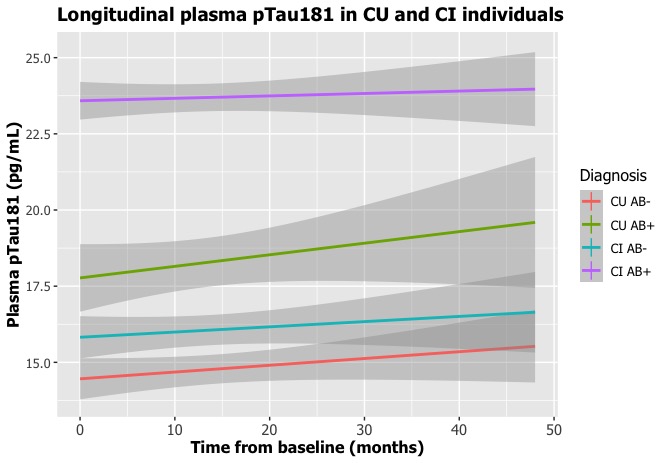


Supplementary Figure 3: Longitudinal plasma pTau181 changes among cognitively unimpaired and cognitively impaired individuals, stratified by Aβ status.

***Supplementary tables***:

| Characteristics | CU | CI |
| --- | --- | --- |
| Number of subjects | 354 | 685 |
| Age (mean, SD) in years | 74.59 (6.61) | 73.30 (7.84) |
| Females (n, %) | 188 (53%)^‡^ | 290 (42%)^‡^ |
| MMSE score (mean, SD) | 29.06 (1.18) ^†^ | 27.11 (2.59) ^†^ |
| Plasma pTau181 (mean, SD) | 15.35 (9.73) ^†^ | 19.62 (10.74) ^†^ |
| Aβ status (positive, %) | 72 (20%) ^†^ | 343 (50%) ^†^ |

^‡^Statistical difference between groups (*P* = 0.001).

^†^Statistical difference between groups (*P*<0.001).

Supplementary Figure 3: Longitudinal plasma pTau181 changes among cognitively unimpaired and cognitively impaired individuals, stratified by Aβ status.
